# Development and Relationships Between Phonological Awareness, Morphological Awareness and Word Reading in Spoken and Standard Arabic

**DOI:** 10.3389/fpsyg.2018.00356

**Published:** 2018-04-09

**Authors:** Rachel Schiff, Elinor Saiegh-Haddad

**Affiliations:** ^1^Learning Disabilities Studies, Haddad Center for Dyslexia and Learning Disabilities, School of Education, Bar-Ilan University, Ramat Gan, Israel; ^2^Department of English, Bar-Ilan University, Ramat Gan, Israel

**Keywords:** Arabic, diglossia, linguistic distance, phonological awareness, morphological awareness, word reading accuracy, word reading fluency

## Abstract

This study addressed the development of and the relationship between foundational metalinguistic skills and word reading skills in Arabic. It compared Arabic-speaking children’s phonological awareness (PA), morphological awareness, and voweled and unvoweled word reading skills in spoken and standard language varieties separately in children across five grade levels from childhood to adolescence. Second, it investigated whether skills developed in the spoken variety of Arabic predict reading in the standard variety. Results indicate that although individual differences between students in PA are eliminated toward the end of elementary school in both spoken and standard language varieties, gaps in morphological awareness and in reading skills persisted through junior and high school years. The results also show that the gap in reading accuracy and fluency between Spoken Arabic (SpA) and Standard Arabic (StA) was evident in both voweled and unvoweled words. Finally, regression analyses showed that morphological awareness in SpA contributed to reading fluency in StA, i.e., children’s early morphological awareness in SpA explained variance in children’s gains in reading fluency in StA. These findings have important theoretical and practical contributions for Arabic reading theory in general and they extend the previous work regarding the cross-linguistic relevance of foundational metalinguistic skills in the first acquired language to reading in a second language, as in societal bilingualism contexts, or a second language variety, as in diglossic contexts.

## Introduction

Arabic is a typical case of diglossia (Ferguson, 1959), which is a sociolinguistic context in which speakers within a single speech community simultaneously use two varieties of a language: one for everyday communication and another for formal interactions and writing. Diglossia is a widespread phenomenon (Myhill, 2014). In Arabic, children grow up speaking Spoken Arabic (SpA) for everyday speech at home and in the neighborhood and Standard Arabic (StA) for reading and writing, as well as for formal interaction, as within the classroom (Amara, 1995; Saiegh-Haddad and Henkin-Roitfarb, 2014). While dialects of SpA are different between different nationality-based Arabic-speaking communities (e.g., Versteegh, 1997, 2001), StA is largely uniform across the Arabic-speaking world (Holes, 2004) and shares many linguistic characteristics such as phonology, morphology, syntax, and lexicon. At the same time, all SpA vernaculars are different from StA (Maamouri, 1998).

This linguistic distance traverses all linguistic domains and is remarkable in the phonology and in the lexicon. For instance, a recent study recorded 5-year-olds as they were interacting with each other on a regular kindergarten day. A corpus of about 4,500 different word types were collected and analyzed for their lexical–phonological distance from their equivalent form in StA. The analysis showed that only about 21% of the words in the spoken lexicon of children consisted of identical words, that is, words that maintain an identical lexico-phonological structure in StA, whereas the remaining words were divided almost evenly between cognate words (with overlapping phonological forms in SpA and StA) and completely different unique forms in SpA and StA (Saiegh-Haddad and Spolsky, 2014).

This linguistic distance between SpA and StA was also found to affect children’s phonological processing skills, such as phonological awareness (PA), phonological processing, naming (Saiegh-Haddad, 2003, 2004, 2007; Saiegh-Haddad et al., 2011; Asaad and Eviatar, 2013; Saiegh-Haddad and Ghawi-Dakwar, 2017), children’s lexical and morpho-syntactic skills, such as negation and inflection (Khamis-Dakwar and Froud, 2007; Khamis-Dakwar et al., 2012), and ultimately their reading skills (Saiegh-Haddad, 2005; Saiegh-Haddad and Schiff, 2016; Schiff and Saiegh-Haddad, 2017). These findings indicate difficulty among native-speaking children in constructing accurate and stable phonological representations for linguistic structures that are not within their spoken vernacular, which impacts processing at all linguistic levels. This diglossia effect has been argued to be a central feature of reading development in Arabic (Saiegh-Haddad and Everatt, 2017).

Besides linguistic distance between SpA and StA, reading acquisition in Arabic implicates another important feature. This is the use of diacritics in the Arabic orthography to encode phonological information necessary for reading accuracy. Orthography is defined as a set of principles that define the basic units of the writing system (Perfetti et al., 2007). However, orthographies vary in the nature of mapping of phonemes onto graphemes; a transparent orthography is considered easier to decode than an opaque orthography in which phoneme and letter correspondences are less regular (Schiff, 2012). Vowelization refers to the use of optional diacritics that the orthography employs to represent vowels and other features of word articulation including, in the case of Arabic, null vowelization, consonant gemination, as well as morpho-syntactic markers of case and mood. Saiegh-Haddad and Henkin-Roitfarb (2014) distinguish two systems of optional diacritics in Arabic. The first comprises phonemic diacritics which include the five diacritical marks mapping short vowels, consonant lengthening/doubling, and null vowelization; these diacritical marks can appear on almost all of the letters, and they map meaningful phonemic information about the word lexeme. The second set of diacritics, however, is morpho-syntactic; they appear at the end of the word stem and map syntactic roles and properties including case and mood. Morpho-syntactic inflections have disappeared from all dialects of SpA but have been preserved in StA (Maamouri, 1998). The modal endings of verbs and the case endings of definite nouns consist of the three Arabic short vowels and are orthographically represented using the same phonemic diacritics. However, the case endings of indefinite nouns are phonologically and orthographically distinct from the other diacritical marks (for a comprehensive discussion, see Saiegh-Haddad and Henkin-Roitfarb, 2014).

This two-layered system of optional diacritics results in two orthographies: a voweled orthography largely transparent: regular and consistent (Schmalz et al., 2015) and an unvoweled orthography, which is an abjad (Daniels, 1992) that is phonologically underspecified and maps only the consonants and long vowels of words, but is morphologically regular and maps both the root and the consonantal and long vowel material of word-patterns. Hence, the Arabic orthography may be considered shallow when it is used in its voweled form. However, due to its root and word-pattern morphological structure, and given the fact that all content words abide by a templatic vocalic pattern, it is possible to recover the phonological information encoded by diacritics, and which is missing in unvoweled Arabic, using the word-pattern morphological structure. This yields the unvoweled form morphologically regular and transparent, though it is phonologically underspecified and deep. For instance, the unvoweled orthographic form MTurk is orthographically deep because the short vowel in the first syllable is missing. However, the first consonant /m/ represented by the letter {M} and the long vowel /u:/ represented by the letter {U} (both represented by letters) indicate the word-pattern to the reader and, hence, the missing short vowel, which in this case can only be /a/. The default Arabic orthography is unvoweled, whereas vowelization is used in the teaching of reading as well as in religious and literary texts.

The question of role that diacritical vowelization has in reading in Arabic has been studied extensively. This research shows that diacritics facilitate reading accuracy and comprehension in both poor and skilled readers (Abu-Rabia and Siegel, 1995; Abu-Rabia, 1997a,b, 1998, 2001). More recent research, however, paints a different picture and shows that diacritic vowelization may result only in more accurate reading in the early grades and in reading disabled children (Schiff and Saiegh-Haddad, 2017). Moreover, diacritical vowelization has been found to reduce reading fluency across all grades from childhood to adolescence (Saiegh-Haddad and Schiff, 2016). It is noteworthy that early research on the role of diacritical vowelization did not distinguish between phonemic and morpho-syntactic diacritics, and this might explain some of the mixed patterns of results observed. Vowelization was also found to burden the perception of words (Abdulhadi et al., 2011; Eviatar and Ibrahim, 2014) and to increase the number and duration of eye fixations (Roman and Pavard, 1987).

The co-occurrence of diglossia and vowelization in Arabic, as in many Arabic-script-based orthographies in Africa (Mumin and Versteegh, 2014), provides a unique context for testing the independent and the interactive effect of these two factors on reading across development. It also allows an investigation of the relative role of metalinguistic factors, such as phonological and morphological awareness in reading in the two varieties and in the two orthographies. This is an important question because “vowelization determines the phonological transparency of the orthography that readers deal with, and this might interact with the effect of linguistic distance on reading in different age groups” (Saiegh-Haddad and Schiff, 2016, p. 4). Moreover, voweled Arabic is phonologically transparent but voweled Arabic is morphologically transparent. This suggests possible differences in the relative role of phonological versus morphological awareness skills to reading in the two orthographies across development (Saiegh-Haddad and Taha, 2017).

The role of linguistic distance and diacritic vowelization on Arabic reading has been the focus of many research studies (Ibrahim, 1983; Eviatar and Ibrahim, 2014; Schiff and Saiegh-Haddad, 2017). Rather than addressing this question, the current study focuses on the development and cross-variety relationships across the school years. It examines the development of and the relationships between PA, morphological awareness, and word reading (voweled and unvoweled) in Arabic across the school grades. The study also probes whether metalinguistic skills in SpA, the variety that children acquire first and use for everyday speech, predicts their reading in Standard Arabic, the language of literacy and which they usually acquire later. This question is critical because children first learn to read in a language that they do not speak, and therefore they graft StA reading on the oral language skills they have developed in SpA. The question of whether literacy-related skills that they develop in SpA predict their reading success in StA is important, and it has significant practical implications for instruction and assessment. Moreover, reading instruction in Arabic is agnostic of the linguistic distance between SpA and StA, and it often does not capitalize on children’s metalinguistic and other literacy-related skills in SpA to leverage acquisition of reading in StA (Saiegh-Haddad and Everatt, 2017). It is to be remembered that SpA, as it is used in the context of this study, refers to linguistic structures (phonemes, morphemes, and words) that are used both in SpA and in StA, rather than structures that are only used within SpA and which are not encoded in Arabic orthography.

### Phonological and Morphological Awareness Skills in Reading

Phonological awareness (PA) refers to one’s awareness of, and access to, the sound structure of oral language (Adams, 1990; Torgesen et al., 1997). The role of PA in learning to read has been strongly established in both L1 and L2 (Wagner and Torgesen, 1987; August and Shanahan, 2006; Saiegh-Haddad and Geva, 2008) including in Arabic (al-Mannai and Everatt, 2005; Elbeheri and Everatt, 2007; Farran et al., 2012; Saiegh-Haddad and Taha, 2017). To acquire the alphabetic principle and to accurately map grapheme to phonemes, the child must acquire the ability to analyze, synthesize, and manipulate constituent phonemes (Stanovich, 1986). Indeed, research shows that PA is concurrently correlated with reading performance and also predicts future reading ability (Casalis and Colé, 2009). Specifically, kindergartners with strong PA skills make better progress in reading than children with low PA skills (Gough and Tunmer, 1986; Wagner and Torgesen, 1987; Torgesen et al., 1997). Moreover, individual differences in kindergartners’ PA explain variations in reading abilities from kindergarten through fourth grade (Wagner et al., 1997). This suggests a causal connection between PA and individual differences in the ability to decode words and non-words (cf. Share, 1995).

Although phonological recoding at the level of grapheme-to-phoneme decoding is essential for the development of successful reading, alphabetic orthographies represent two layers of language: phonemes and morphemes. Therefore, awareness of morphemes should contribute to reading development in an alphabetic orthography besides awareness of phonemes (Verhoeven and Perfetti, 2003; Saiegh-Haddad and Geva, 2008). Moreover, typological differences between languages and orthographies are often noticeable in the morphological structure with some orthographies, like English, being scarce morphologically (often not depicting word-internal morphological structure), as against other orthographies like Arabic and Hebrew (Semitic languages) which are very rich morphologically and where the majority of words encode an internal morphological structure. Because writing systems are isomorphic (Verhoeven and Perfetti, 2003), that is they represent the morphological structure though to different degrees of explicitness, these differences in morphological richness are often also reflected in the orthographic structure of words in different languages and this, in turn, has repercussions for morphological awareness development and processing in reading and spelling in different languages (e.g., Bryant et al., 1999; Assink et al., 2000; Ravid and Bar-On, 2005; Gillis and Ravid, 2006; Saiegh-Haddad and Geva, 2008; Saiegh-Haddad, 2013; Taha and Saiegh-Haddad, 2016, 2017; Saiegh-Haddad and Taha, 2017). For example, In the Arabic writing system, the morphemic units in words are not concatenated as is the case in English or French. Because the root is inserted within fixed slots in the pattern, the word structure is represented and perceived differently from non-Semitic languages (Boudelaa, 2014).

Morphological awareness is defined as the ability to reflect on and manipulate the constituent morphemes of words, the smallest meaningful word units (Carlisle, 1995). Research shows that readers develop awareness of the morphological structure and demonstrate the understanding of morphological associations between words (Carlisle, 1995; Bryant et al., 2000; McBride-Chang et al., 2003). This ability has been shown to contribute to decoding, word recognition, and reading comprehension (Deacon and Kirby, 2004; Nagy et al., 2006; Ravid and Schiff, 2006a,b; Schiff et al., 2011). However, recent views suggest a two-way interaction between morphological awareness, reading comprehension (Perfetti et al., 2005; Deacon et al., 2014), and word reading accuracy (Deacon et al., 2013). As such, morphological awareness assists children comprehend written texts both through a direct relationship with reading comprehension and through a more indirect relationship by helping them to encode individual words, which, in turn, promote the skills of reading comprehension (Deacon et al., 2014).

In the Arabic writing system, the two basic morphemic units in words: the root and the word-pattern are not linearly concatenated, as is the case in English or French. Yet, they are regularly represented in the letter structure of the word as explained above. The root is a strong semantic entity and is a constituent of the stem of almost all content words in Arabic. All this implies the salience of the root in processing Semitic Arabic. In fact, the root and the word-pattern appear to be central to the way that words are organized in the Arabic lexicon (Boudelaa, 2014). It also appears to be implicated in early reading and spelling in Arabic (Saiegh-Haddad, 2013; Taha and Saiegh-Haddad, 2016, 2017), to be impaired in reading disabled readers (Abu-Rabia et al., 2003; Saiegh-Haddad and Taha, 2017), and to be used as a compensatory mechanism among reading disabled to aid their phonological deficits (Saiegh-Haddad and Taha, 2017).

Given the morphological richness of Arabic (Saiegh-Haddad and Henkin-Roitfarb, 2014) and the proliferation of morphology in the linguistic and orthographic representation of the word and, in turn, in word reading and spelling in Arabic (Abu-Rabia et al., 2003; Abu-Rabia, 2007; Saiegh-Haddad, 2013, 2017; Taha and Saiegh-Haddad, 2016, 2017; Saiegh-Haddad and Taha, 2017; Tibi and Kirby, 2017), the study of morphological processing in Arabic is highly warranted. Moreover, because there are differences between SpA and StA in inflectional morphology, with some StA inflectional categories not encoded in SpA, such as dual and plural feminine verbal forms, and in derivational morphology, with some StA word-patterns not used in SpA (Saiegh-Haddad and Henkin-Roitfarb, 2014), it is critical to study the role of morphological awareness in reading Arabic, as well as the relevance of SpA morphology in particular to reading in StA (Roman et al., 2009; Wolter et al., 2009).

The goal of the present study is to examine the development of PA and morphological awareness in SpA and StA separately. It further explores word reading in SpA among Arabic-speaking children compared with their StA word reading with the objective of probing quantitative differences between these abilities in the two language varieties and their relation to StA reading ability. The question of whether the children’s SpA metalinguistic awareness and word reading show different developmental trajectories, and whether they show different patterns of relationships with reading in StA, has never been studied. Moreover, earlier research never addressed this question in the reading of voweled versus unvoweled words separately. These questions are critical given the fact that children are taught to read in StA whereas the language they master and naturally use is SpA, a language that differs from SpA in all linguistic domains including in the phonological and in the morphological structure. This developmental examination is also warranted given the phonological transparency of voweled Arabic, yet the morphological transparency of unvoweled Arabic. This might impact the relevance of phonological versus morphological awareness to reading in different grades an in voweled versus unvoweled Arabic.

### The Current Study

To date, only one study has investigated the effect of diglossia on the development of reading skills in voweled and unvoweled SpA and StA among typically developing readers (Saiegh-Haddad and Schiff, 2016) and another tested this question in reading disabled children (Schiff and Saiegh-Haddad, 2017). The results of these studies underscore the role of diglossia and diacritical vowelization in understanding reading development in Arabic. The present study extends this previous investigation to the effect of diglossia on the skills of PA and morphological awareness as well. A second goal of this study is to investigate the contribution of PA and morphological awareness in SpA to StA word reading. We hypothesized that children would demonstrate a higher level of SpA than StA PA and morphological awareness because they experience greater exposure to SpA. We also predicted that SpA PA and morphological awareness would consistently provide a unique contribution to StA word reading. Finally, it was predicted that differences between SpA and StA phonological and morphological awareness would decrease with development, due to increasing exposure to StA through schooling. Moreover, the role of PA in predicting reading was expected to decrease with development, whereas the role of MA was expected to increase. It is noteworthy that SpA, as it is used in the context of this study, refers to linguistic structures (phonemes, morphemes, and words) that are used both in SpA and in StA, rather than to unique SpA structures that are not encoded in Arabic written language.

## Materials and Methods

### Participants

A total of 100 students participated in the study: 20 second graders (age: *M* = 7;7, *SD* = 3.00 months), 20 fourth graders (age: *M* = 9;6, *SD* = 4.00), 20 sixth graders (age: *M* = 11;6, *SD* = 3.72), 20 eighth graders (age: *M* = 13;6, *SD* = 4.10), and 20 tenth graders (age: *M* = 15;5, *SD* = 3.08). There were 10 female and 10 male students in each grade level. All participants were native speakers of a local dialect of Palestinian Arabic spoken in the north of Israel and were sampled from two public schools in the north school district with an officially ranked middle socioeconomic background. No participant had reported neurological, language, or psycho-educational difficulties. Data collection took place during the winter–spring of 2016. Official authorization by the chief scientist of the Ministry of Education, as well as by Bar-Ilan University ethics committee was obtained. Written parental consent was obtained for all children participating in the study.

### Materials

#### Phonological Awareness

Two sets of PA tasks were developed: one in SpA and another in StA. SpA tasks used phonological structures (phonemes and syllabic structures) that are used in both StA and the SpA vernacular used by the children, whereas StA tasks targeted phonological structures that are used only in StA. Phonological structures (mainly phonemes) that are used only in SpA were not targeted because these may not have a conventional orthographic representation, namely, a grapheme that represents them in the written language. Two tasks per language variety (SpA and StA) were developed: full phoneme segmentation equally targeting initial, final, and medial phonemes (*N* items = 15 per language variety; Cronbach alpha: SpA 0.89 and StA 0.90) and phoneme deletion (*N* items = 15 per language variety; Cronbach alpha: SpA 0.87 and StA 0.90). One score was assigned for completing each item correctly and a zero score for any kind of error. No partial scores were assigned. The PA score is a total score obtained on both the phoneme segmentation and the deletion tasks per each language variety separately. It is important to note that the correlation between the two tasks was high, and therefore we used a composite score.

#### Morphological Awareness

Two sets of morphological awareness tasks were developed: one in SpA (*N* items = 40) and another in StA (*N* items = 40; equal number of inflectional and derivational morphology) SpA tasks targeted morphological units that are used in both StA and the SpA vernacular used by the children (Cronbach’s alpha: inflection 0.82, derivation 0.91), whereas StA tasks targeted morphemes that are used only in StA (Cronbach’s alpha: inflection 0.81, derivation 0.90). The morphological tasks employed the *Word Analogy* format adapted from Nunes et al. (1997). This is an oral task in which students are required to produce a missing word that follows a certain pattern from a given set of word pairs. For example, run: runs; walk: _____ (walks). For example, for testing inflectional morphology, the following item was used: 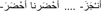 He brought – We brought; He achieved, We ________, and for testing derivational morphology, the following item was used: 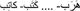 Wrote-writer; Ran-…… .

#### Word-Reading Measures

Twelve different word-level reading tasks were constructed that measured accuracy and fluency of reading SpA and StA voweled and unvoweled real and pseudowords (*N* = 110 items per task). For the real word reading tasks, we selected SpA words with identical word forms in StA and SpA (e.g., /*ba:b*/ “door,”/*na:m*/ “slept,”/*bi:Ɂa*/ “environment,” and /*Ɂistaɣall*/ “exploited”) and StA words that exist in StA but not in SpA. The latter word type comprised cognate words, which have similar forms in StA and SpA (e.g., StA /*θalƷ*/- SpA /*taliƷ*/ “snow”), and unique words, which are used in StA but not in SpA (StA /*Ʒara:*/- SpA /*rakad*/ “ran”). Words were matched on phonemic length (three to 12 phonemes), syllabic length (one to five syllables), orthographic length (three to ten letters), morphological structure (one to four morphemes), and on word familiarity/frequency as determined by 10 Arabic language experts based on a five-point scale. StA words across tasks were also matched on the type of the StA linguistic structure they encoded (phoneme, syllable structure, and morphological template). Words within tasks progressed in linguistic complexity and frequency. Separate lists of words were presented in the voweled (phonemic diacritics only) and unvoweled orthography. None of the unvoweled words were homographic or could be read in more than one way based on the specific diacritics that were missing. SpA and StA pseudowords were derived from the voweled words by changing one or two letters in the word while ensuring that the pseudowords abided by SpA and StA phonotactic rules, respectively (e.g., SpA /*xama:d*/ from the word /*šama:l*/ “north” and StA /*«ala:b*/ from the word /*«aha:b*/ “going”). Pseudowords across tasks were matched on phonemic length, orthographic length, and syllabic complexity.

### Procedure

Tasks were administered individually by a Ph.D. student, a native speaker of the dialect targeted in this study. Task administration took place in a quiet room at school. The order of administration of the fluency and accuracy sets was counterbalanced, and tasks within each set were intermixed. Task administration started after three practice trials. To measure accuracy, participants were instructed to read the list of words as accurately as possible and testing was discontinued after five consecutive errors. Accuracy was calculated as the number of words read accurately out of the total number of words (110 items). To measure fluency, participants were instructed to read the list of words as accurately and as quickly as possible, and testing was discontinued after 1 min. Reading fluency was calculated as the number of words read accurately in the first 45 s of testing. Data collection took place in May, 2 months before the end of the school year. Analysis participants’ performance on the reading accuracy tasks showed satisfactory Cronbach’s alpha reliability levels exceeding 0.7.

## Results

### Phonological and Morphological Awareness in SpA and StA Tests

In order to examine performances on the phonological and morphological awareness tests, two 5×2 repeated measures analyses of variance (ANOVA) were conducted, with grade (second, fourth, sixth, eighth, and tenth) as the between-subject variable, and language variety (SpA and StA) as the within-subject variable: one analysis for the PA and one for the morphological awareness.

#### Phonological Awareness in SpA and StA Tests

The main effect of language variety was found to be significant, indicating greater performance on the SpA than on the StA test. The main effect of group was also significant, indicating that the students in the higher grades performed better than those in the lower grades (**Table 1**).

**Table 1:** Means (and SD) of phonological, morphological, and morpho-syntax awareness in SpA and StA tests by grade.

Language variety								
		*SpA*		*StA*				
Tests	Grades	*M*	*SD*	*M*	*SD*	*F* grade	*F* language variety	*F* interaction
Phonological awareness	Second	47.48	28.43	25.82	20.92			
	Fourth	69.00	13.16	52.33	9.18			
	Sixth	60.83	15.93	58.17	17.59			
	Eighth	82.22	12.06	80.93	17.59			
	Tenth	82.54	9.66	82.00	10.40	30.11^∗∗∗^	50.69^∗∗∗^	13.45^∗∗∗^
Morphological awareness	Second	73.25	16.62	56.06	16.29			
	Fourth	90.62	7.82	76.00	13.40			
	Sixth	91.00	10.34	81.82	15.65			
	Eighth	96.50	4.17	94.87	4.23			
	Tenth	97.25	4.21	92.56	7.47	29.67^∗∗∗^	124.62^∗∗∗^	11.87^∗∗∗^

*^∗∗∗^p < 0.001.*

**FIGURE 1:**
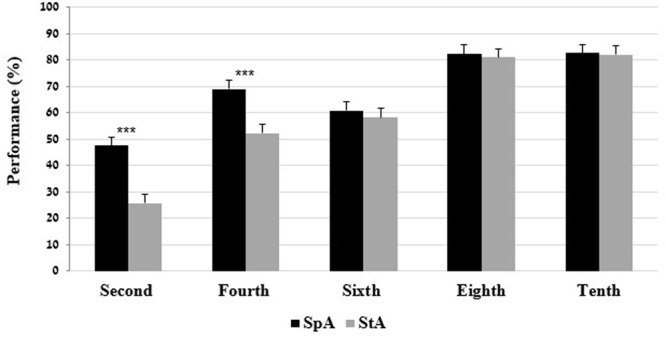
Mean (and SE) performance on the phonological awareness test by grade and language variety. ^∗∗∗^*p* < 0.001.

The two-way interaction of grade × language variety was significant on the PA test. Bonferroni analysis indicated that the second and fourth grades performed better on the SpA compared to the StA. No significant differences between the performances on the SpA tests and the StA tests were found in the sixth, eighth, and tenth grades (**Figure 1**).

#### Morphological Awareness in SpA and StA Tests

The main effect of language variety was found to be significant, indicating greater performance on the SpA than on the StA test. The main effect of group was also significant, indicating that the students in the higher grades performed better than those in the lower grades (**Table 1**).

**FIGURE 2:**
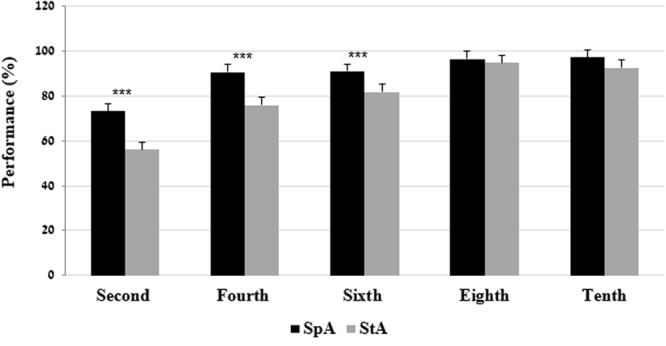
Mean (and SE) performance on the morphological awareness test by grade and language variety. ^∗∗∗^*p* < 0.001.

The two-way interaction of grade × language variety was significant on the morphological awareness test. Bonferroni analysis indicated that the second, the fourth, and the sixth grades performed better on the SpA compared to the StA. No significant differences between the performances on the SpA tests and the StA tests were found in the eighth and tenth grades (**Figure 2**).

### Reading Accuracy and Fluency in Voweled and Unvoweled SpA and StA Tests

In order to examine performances on the reading accuracy and fluency tests, four 5×2 repeated measures analyses of variance (ANOVA) were conducted, with grade (second, fourth, sixth, eighth, and tenth) as the between-subject variable, and language variety (SpA and StA) as the within-subject variable: two analyses for the performances on the accuracy tests and two analyses for the performances on the fluency tests. For the reading accuracy tests, we also conducted two 5×2 repeated measures analyses of variance (ANOVA) for the item level, with grade as the within-participants variable and language variety as the between-participants variables.

#### Reading Accuracy in Voweled and Unvoweled SpA and StA Tests in the Subject Level

The main effects of language variety were found to be significant, indicating greater performance on the SpA than on the StA on the two reading accuracy tests. The main effects of group were also significant, indicating that the students in the higher grades performed better than those in the lower grades (**Table 2**).

**Table 2:** Means (and SD) of reading accuracy and fluency in voweled and unvoweled SpA and StA tests by grade.

Language variety								
		*SpA*		*StA*				
Tests	Grades	*M*	*SD*	*M*	*SD*	*F* grade	*F* language variety	*F* interaction
Phonological awareness	Second	47.48	28.43	25.82	20.92			
	Fourth	69.00	13.16	52.33	9.18			
	Sixth	60.83	15.93	58.17	17.59			
	Eighth	82.22	12.06	80.93	17.59			
	Tenth	82.54	9.66	82.00	10.40	30.11^∗∗∗^	50.69^∗∗∗^	13.45^∗∗∗^
Morphological awareness	Second	73.25	16.62	56.06	16.29			
	Fourth	90.62	7.82	76.00	13.40			
	Sixth	91.00	10.34	81.82	15.65			
	Eighth	96.50	4.17	94.87	4.23			
	Tenth	97.25	4.21	92.56	7.47	29.67^∗∗∗^	124.62^∗∗∗^	11.87^∗∗∗^

*^∗∗^p < 0.01, ^∗∗∗^p < 0.001.*

**FIGURE 3:**
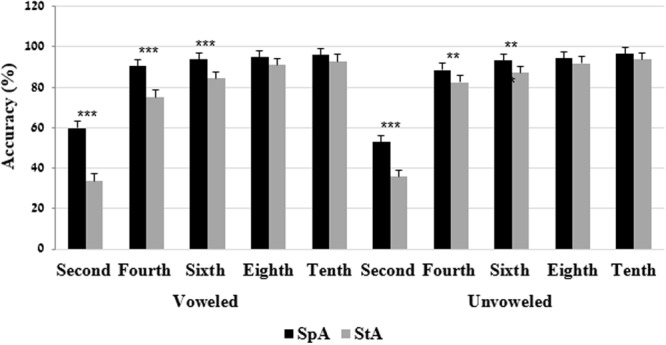
Mean (and SE) performance on the accuracy test by grade and language variety. ^∗∗^*p* < 0.01, ^∗∗∗^*p* < 0.001.

The two-way interactions of grade × language variety were significant on both voweled and unvoweled reading accuracy tests. Bonferroni analyses on both tests indicated that the second, the fourth, and the sixth grades performed better on the SpA compared to the StA. No significant differences between the performances on the SpA tests and the StA tests were found in the eighth and tenth grades (**Figure 3**).

#### Reading Accuracy in Voweled and Unvoweled SpA and StA Tests in the Item Level

The main effects of language variety were found to be significant on both voweled and unvoweled reading accuracy tests [*F*(1,10895) = 1074.62, *p* < 0.001, $\eta_{p}^{2}$ = 0.09 and *F*(1,10895) = 1174.48, *p* < 0.001, $\eta_{p}^{2}$ = 0.30, respectively], indicating greater performance on the SpA than on the StA on the two reading accuracy tests. The main effects of group were also significant on both accuracy tests [*F*(4,10895) = 1028.92, *p* < 0.001, $\eta_{p}^{2}$ = 0.27 and *F*(4,10895) = 524.97, *p* < 0.001, $\eta_{p}^{2}$ = 0.05, respectively], indicating that the students in the higher grades performed better than those in the lower grades. The two-way interactions of grade × language variety were significant on both voweled and unvoweled reading accuracy tests [*F*(4,10895) = 80.72, *p* < 0.001, $\eta_{p}^{2}$ = 0.03 and *F*(4,10895) = 29.16, *p* < 0.001, $\eta_{p}^{2}$ = 0.01, respectively]. Bonferroni analyses on both tests indicated that the second, the fourth, and the sixth grades performed better on the SpA compared to the StA. No significant differences between the performances on the SpA tests and the StA tests were found in the eighth and the tenth grades.

#### Reading Fluency in Voweled and Unvoweled SpA and StA Tests

The main effects of language variety were found to be significant, indicating greater performance on the SpA than on the StA on the two reading fluency tests. The main effects of group were also significant, indicating that the students in the higher grades performed better than those in the lower grades (**Table 2**).

**FIGURE 4:**
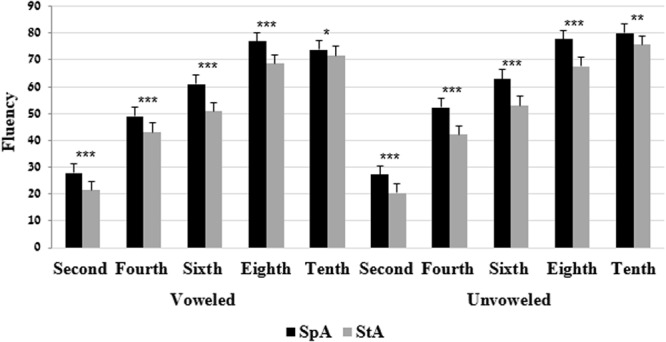
Mean (and SE) performance on the fluency test by grade and language variety. ^∗^*p* < 0.05, ^∗∗^*p* < 0.01, ^∗∗∗^*p* < 0.001.

The two-way interactions of grade × language variety were significant on both voweled and unvoweled reading fluency tests. Bonferroni analyses indicated that on both the voweled and the unvoweled StA and SpA fluency tests, all the five grades performed better on the SpA compared to the StA (**Figure 4**).

### Contribution of Participants’ Grade and the Performances on the SpA Phonological and Morphological Awareness to the Prediction of the Performances on the Reading Accuracy and Fluency in the StA Tests

Pearson correlations were conducted in order to examine the correlations between the performances on the reading accuracy and fluency in the StA tests and the performances on the SpA phonological and morphological awareness tests (**Table 3**).

**Table 3:** Correlations between the performance on the reading accuracy and fluency in the StA tests and the performance on the SpA phonological, morphological, and morpho-syntax awareness tests.

		Phonological awareness	Morphological awareness
Accuracy	Voweled StA	0.70^∗∗∗^	0.75^∗∗∗^
	Unvoweled StA	0.66^∗∗∗^	0.75^∗∗∗^
Fluency	Voweled StA	0.62^∗∗∗^	0.68^∗∗∗^
	Unvoweled StA	0.60^∗∗∗^	0.66^∗∗∗^

*^∗∗∗^p < 0.001.*

**Table 3** indicates significant positive correlations that as the performances on the SpA phonological and morphological awareness tests increase, the performances on the reading accuracy and fluency in the StA tests increase, respectively.

In order to examine the contribution of the grade and the performances on the SpA phonological and morphological awareness tests to the prediction of the performances on the reading accuracy and fluency in the StA tests, we conducted four hierarchical regression analyses, two analyses for the prediction of the performance on the voweled and unvoweled reading accuracy tests and two analyses for the prediction of the performance on the voweled and unvoweled reading fluency tests. In the first step, the background characteristic – grade was entered. In the second step, the performance on the PA test were entered, and in the third step, the performance on the morphological awareness test were entered. We entered the performance on the morphological awareness test in the third step in order to examine whether the performances would contribute significantly to the explained variances of the performances on the accuracy and fluency in the StA tests, beyond the background characteristic and the performance on the PA test (**Table 4**).

**Table 4:** Results of mixed regressions for the performance on StA reading accuracy and fluency tests by participants’ grade and SpA phonological, morphological and morpho-syntax awareness tests.

Dependent variables	Steps	Independent variables	*B*	SE *B*	β	*t*	*R*^2^	Δ*R*^2^
Voweled StA accuracy	**1**	Grade	6.70	0.58	0.76	11.54^∗∗∗^	0.576^∗∗∗^	0.576^∗∗∗^
	**2**	Grade	4.74	0.60	0.54	7.91^∗∗∗^		
		Phonological awareness	0.47	0.08	0.40	5.92^∗∗∗^	0.689^∗∗∗^	0.113^∗∗∗^
	**3**	Grade	3.95	0.59	0.45	6.72^∗∗∗^		
		Phonological awareness	0.24	0.09	0.20	2.55^∗^		
		Morphological awareness	0.66	0.16	0.34	4.13^∗∗∗^	0.736^∗∗∗^	0.047^∗∗∗^
Unvoweled StA accuracy	**1**	Grade	6.28	0.59	0.73	10.57^∗∗∗^	0.533^∗∗∗^	0.533^∗∗∗^
	**2**	Grade	4.50	0.63	0.52	7.08^∗∗∗^		
		Phonological awareness	0.43	0.08	0.38	5.09^∗∗∗^	0.631^∗∗∗^	0.098^∗∗∗^
	**3**	Grade	3.59	0.61	0.42	5.84^∗∗∗^		
		Phonological awareness	0.16	0.10	0.14	1.66		
		Morphological awareness	0.75	0.17	0.40	4.53^∗∗∗^	0.696^∗∗∗^	0.065^∗∗∗^
Voweled StA fluency	**1**	Grade	6.29	0.40	0.84	15.54^∗∗∗^	0.711^∗∗∗^	0.711^∗∗∗^
	**2**	Grade	5.36	0.46	0.72	11.74^∗∗∗^		
		Phonological awareness	0.22	0.06	0.23	3.70^∗∗∗^	0.747^∗∗∗^	0.036^∗∗∗^
	**3**	Grade	4.93	0.47	0.66	10.56^∗∗∗^		
		Phonological awareness	0.10	0.07	0.10	1.31		
		Morphological awareness	0.36	0.13	0.22	2.83^∗∗^	0.767^∗∗∗^	0.019^∗∗^
Unvoweled StA fluency	**1**	Grade	6.81	0.38	0.88	18.08^∗∗∗^	0.769^∗∗∗^	0.769^∗∗∗^
	**2**	Grade	6.13	0.44	0.79	14.05^∗∗∗^		
		Phonological awareness	0.16	0.06	0.16	2.85^∗∗^	0.787^∗∗∗^	0.018^∗∗^
	**3**	Grade	5.72	0.45	0.74	12.82^∗∗∗^		
		Phonological awareness	0.04	0.07	0.04	0.62		
		Morphological awareness	0.34	0.12	0.20	2.82^∗∗^	0.803^∗∗∗^	0.016^∗∗^

*^∗∗^p < 0.01, ^∗∗∗^p < 0.001.*

**Table 4** indicates that the performances on the SpA PA tests added significantly 11.3 and 9.8% beyond the participants’ grade to the prediction of the performance on the voweled and unvoweled StA accuracy tests (*p*s < 0.001) and 3.6 and 1.8% to the prediction of the performance on the voweled and unvoweled StA fluency tests (respectively). The positive beta coefficient of participants’ grade and the performances on the SpA PA tests to the prediction of the performances on the voweled and the unvoweled StA accuracy and fluency tests indicated that as the grade and the performances on the SpA PA tests increase, the performances on the voweled and the unvoweled StA accuracy and fluency tests increase, respectively.

In the third step, the performances on the SpA morphological awareness tests added significantly 4.7 and 6.5% beyond the participants’ grade and the performance on the PA test to the prediction of the performance on the voweled and unvoweled StA accuracy tests (*p*s < 0.001) and 1.9 and 1.6% to the prediction of the performance on the voweled and unvoweled StA fluency tests (*p*s < 0.01, respectively). The positive beta coefficient of the performances on the SpA morphological awareness tests to the prediction of the performances on the voweled and the unvoweled StA accuracy and fluency tests indicated that as the performances on the SpA morphological awareness tests increase, the performances on the voweled and the unvoweled StA accuracy and fluency tests increase, respectively.

Despite the low contribution of the performance on the morphological awareness test in the third step of the regression analyses, it should be noted that the significant contribution of the performance on the PA test to the prediction of the performance on the voweled and unvoweled StA accuracy and fluency tests is moderate and becomes non-significant while the performance on the morphological awareness was entered in the third step.

### Mixed Effects Modeling

Using mixed effects’ modeling indicated that the estimates for the fixed effect of the item level were decreased in 0.0035 and 0.0033 points per item for the accuracy in voweled and unvoweled SpA and StA tests, *p* = 0.11 and *p* = 0.12, respectively. Mixed effects’ modeling analysis for the variance of the item level analysis indicated that the estimates were increased in 0.000019 and 0.000016 points per item for the accuracy in voweled and unvoweled SpA and StA tests, *p* = 0.16 and *p* = 0.15, respectively.

## Discussion

The present study addressed PA, morphological awareness, and word reading in diglossic Arabic by investigating the development of these metalinguistic skills in SpA and StA, and examining the relationship between metalinguistic skills in SpA and reading in StA for both voweled (transparent) and unvoweled (opaque) words. The study also examined children from different age groups in the school system, thus providing a developmental point of view on the link between metalinguistic skills in general, and in relation to voweled and unvoweled word reading in Arabic.

Specifically, the first goal of the study was to explore performance differences on phonological and morphological awareness as well as word reading tasks among Arabic-speaking children, by comparing these tasks in SpA and StA. The examination of PA, morphological awareness, and word reading accuracy and fluency tasks yielded different results. The results showed an initial discrepancy in the second and fourth grades in PA and morphological awareness in SpA and StA words, in favor of the former, that was eliminated in the sixth and eighth grades for the two tasks, respectively. The rather quick improvement in PA compared to morphological awareness might relate to learner’s gradual development in understanding the complex relations of form and meaning (Carlisle, 2010). This development of morphological awareness is effectively observed in first-, third-, and fifth-grade students. Previous research shows a significant increase between the first and fifth grades in the number of derived words that students’ correctly defined (Anglin et al., 1993). These findings provide additional support for the claim that at the beginning of the reading acquisition process, children rely heavily on the phonological information that vowelization provides in order to decode words successfully. As readers acquire sufficient mastery in reading skills, the need decreases for vowelization to accurately decipher words.

The current study also showed that metalinguistic awareness in SpA was consistently higher than that in StA. These findings conform with reported evidence and they offer further evidence for the *Linguistic Affiliation Constraint* (Saiegh-Haddad, 2007) which was developed based on phonological processing research and predicts that linguistic affiliation with SpA versus StA has an effect on linguistic processing in Arabic such that any SpA structure will be easier to access and operate on than its parallel StA structure. The results also support the *diglossia effect* (Saiegh-Haddad, 2017), which is a processing advantage for SpA over StA structures and which is expected to emerge on any reading and metalinguistic awareness task that requires access to and/or operation on linguistic representation. The argument is that in as much as linguistic representation for StA structures is weak, given reduced experience with and practice of StA lexicon, phonology and grammar (Saiegh-Haddad et al., 2011) operations on such representations will suffer (Saiegh-Haddad, 2017).

With respect to reading, this study suggests a number of differences in the development of SpA and StA word reading skills. The results reveal that both word reading accuracy and fluency in Arabic are higher for SpA words than StA words. This advantage for SpA words over StA words was found to persist across development and to surface in the reading of both voweled and unvoweled words. This finding is cardinal, indicating the long-term impact of diglossia and its interplay with orthographic depth. Given the fact that the Arabic orthography, at least in its voweled form, is highly transparent, the results underscore the importance of another factor in word reading development in Arabic. This factor might be diglossia and the linguistic distance between SpA and StA word morphology. This finding might also be explained by the lack of efficiency in basic phonological and morphological processing skills in StA that might clarify some of the possible mechanisms by which diglossia might affect word reading accuracy and fluency in Arabic.

With respect to the role of vowelization in explaining differences in word reading accuracy and fluency skills between SpA and StA, the results showed that the gap in reading accuracy between SpA and StA was evident in the case of both unvoweled and voweled words. While it closes up in the eighth grade in reading accuracy of SpA voweled words, in the case of StA voweled words, the differences vanish when readers reach the tenth grade. In the fluency measures, the gap between unvoweled SpA and StA words remains in all grade levels we checked. This finding coincides with the prediction that differences between SpA and StA should be smaller when reading transparent words than opaque ones. Thus, in reading vowelized words, where successful decoding merely requires grapheme–phoneme correspondence, children reached a similar fluency level in SpA and StA. On the other hand, when reading unvoweled words, children reading StA words encountered greater difficulty in rapidly reading the words. Hence, Arabic-speaking children still seem to rely heavily on simple decoding skills while reading StA. While reading voweled words in Arabic is compatible with a heavy reliance on alphabetic mechanisms in reading and with high rates of reading accuracy (Seymour et al., 2003), this study indicates that the role of vowelization for building reading ability still remains essential in reading StA words.

The second goal of this study was to explore the connection between PA, morphological awareness in SpA, and word reading in StA. Examining these three aspects of language provided an opportunity to clarify the contribution of metalinguistic skills in SpA to StA word reading, as these aspects provide an opportunity to consider answers related to questions such as the following: what aspect is most important to be included in SpA intervention programs, or at what age is it beneficial to provide preventative instruction? Results of this study indicate that in addition to the expected role of SpA PA, SpA morphological awareness plays a cardinal role in StA word reading fluency. This finding is well aligned with the established claim that morphological awareness contributes to school-age students’ performance reading and spelling words or pseudowords (Abu-Rabia et al., 2003; Abu-Rabia, 2007; Saiegh-Haddad, 2013; Taha and Saiegh-Haddad, 2016, 2017). Similar findings have been reported in English (e.g., Deacon and Kirby, 2004; Carlisle and Stone, 2005; Nunes et al., 2006), French (e.g., Casalis and Louis-Alexandre, 2000), Dutch (e.g., Assink et al., 2000), Chinese (e.g., Ku and Anderson, 2003; Chung and Hu, 2007), and Hebrew (Schiff and Lotem, 2011) – to name a few.

In addition to demonstrating a significant contribution of phonological and morphological awareness to reading in Arabic, the current results are particularly interesting given the design of the current study in which phonological and morphological skills were tested in SpA but reading was naturally conducted in StA. The evidence that SpA phonological and morphological awareness predicts StA reading supports earlier evidence of the role of metalinguistic awareness in L1 I predicting reading in L2 (August and Shanahan, 2006). Moreover, they extend previous evidence to diglossic setting like Arabic and supports the conception that it is possible to enhance reading in StA by raising awareness of SpA linguistic structures and without burdening the child with unfamiliar StA linguistic structures. Moreover, SpA and StA metalinguistic awareness tasks might tap not only into awareness of phonology or morphology but also of quality of phonological and morphological representations (Saiegh-Haddad, 2017) making these tasks less valid as measures of metalinguistic awareness.

Our findings, together with those of several previous studies, seem to suggest that morphological awareness is more a language- or variety-specific construct (Pasquarella et al., 2011; Luo et al., 2014). In other words, whether or not transfer from one variety to another would occur is largely influenced by similarities or differences in the morphological features of the two languages. Despite the fact that StA and SpA partly share word structures, StA morphological awareness requires operation on StA morphological structures. Inasmuch as these are low in quality, awareness of these structures is expected to suffer (Saiegh-Haddad, 2017), and therefore, transfer of reading skills between the two varieties is quite limited. It is possible that utilization of SpA structures in StA encoding depends on the demand of reading in StA. Perhaps, reading StA requires PA, whereas reading SpA requires more morphological awareness. In other words, reading StA might benefit more from recognizing inflections as a unit in reading compared with readers of SpA who might benefit more from phonemic segmentation.

The present data support previous reports of the link between diglossia, linguistic skills, and reading ability (Saiegh-Haddad and Schiff, 2016). The findings assert that children who grow up in diglossic context enter school with weaker phonological and morphological skills for the written language (StA) than for the language they use in everyday speech (SpA), and this has cascading consequences on the development of reading skills in general, and reading fluency. It appears that morphological awareness instruction in SpA might provide a stronger foundation for StA word-reading instruction than PA instruction or the standard literacy instruction. Further, in light of previous studies reporting either a direct (Deacon et al., 2014) or indirect (Kieffer and Box, 2013) impact that morphological awareness has on reading comprehension, it would be beneficial to wrap morphological awareness into the Arabic language arts curriculum as preventative treatment that would compensate for the deficits caused by diglossia. A next step, which would have both theoretical and applied values, would be to carry out intervention studies to test further the causal hypothesis and promote the use of developmental research in teaching (Taha and Saiegh-Haddad, 2016, 2017).

It is important to note some directions for further study. First, the present study focused only on phonological and morphological awareness skills as well as reading skills among children from mid-high socio-economic background. Because this study found evidence for differences in these skills, future research should examine the phonological and morphological awareness abilities as well as reading skills among children of different socioeconomic backgrounds, to explore whether the gaps found in this study increase among children from low socio-economic background. Second, the current study is cross-sectional in nature, thus in order to clarify whether the differences in the phonological and morphological awareness as well as reading abilities of children across grades indeed indicate a different developmental pattern, future studies should employ a longitudinal design and follow the same group of children over grades. Finally, future studies could potentially extend the investigation to other diglossic contexts.

## Author Contributions

All authors listed have made a substantial, direct and intellectual contribution to the work, and approved it for publication.

## Conflict of Interest Statement

The authors declare that the research was conducted in the absence of any commercial or financial relationships that could be construed as a potential conflict of interest.
